# Society Meetings

**Published:** 1916-10

**Authors:** 


### SOCIETY MEETINGS

Brief reports and announcements of meetings of societies of Western New York. and those of general scope, are requested from Secretaries. Copy should be on hand the fifteenth of the month. Full transactions will be published at cost of composition.

---

**DOCTOR: Is your Society properly represented here? If not, please bring our standing announcement to the attention of the members.**

---

We have received voluminous reports in galley, from several national societies. Owing to the fact that those especially interested will have access to the papers when published in full in official organs or bound volumes, and that our space is none too great for the local matter more strictly in our province, it has been decided not to publish these reports, valuable as they are.

---

**The Association for the Study of the Internal Secretions** was organized in Detroit during the meeting of the A. M. A. 26 were present at the organization meeting. Nearly 300 applications have been received for charter membership. The annual dues are $5.00, the first period being extended through 1917. Meetings will be held with the A. M. A. The Organization Committee consists of L. F. Barker, Baltimore; Judson Daland, Philadelphia; L. R. De Buys, New Orleans; Emil Goetsch, Baltimore; Henry R. Harrower, Los Angeles; Geo. H. Hoxis, Kansas City; John B. Potts, Omaha. The Treasurer is W. W. Duke, Waldheim Bdg., Kansas City, Mo. A small journal entitled the Link between the Members of the A. S. I. S. has begun issue with Sept. 1916, to be continued monthly. Price 50 cents a year to non-members, included in dues for members.

---

**The Eighth District Branch of the Medical Society of the State of New York** held its eleventh annual meeting in Batavia, Sept. 7, under the presidency of A. T. Lytle of Buffalo. All Officers hold over for the ensuing year as follows: President, Albert T. Lytle, M. D., 200 Lexington Avenue, Buffalo; First Vice-President, Edward Torrey, M. D., Olean; Second Vice-President, William R. Thomson, M. D., Warsaw; Secretary, Lyman C. Lewis, M. D., Belmont; Treasurer, Fitch Van Orsdale, M. D., Belmont. The next meeting will be in Buffalo.

Scientific Session—"Operative Obstetrics."—Dr. Irving W. Potter. Discussion: James E. King, M. D., P. W. Van Peyma, M. D., and Earl P. Lothrop, M. D., Buffalo; Ross G. Loop, M. D., Elmira; and W. Mortimer Brown, M. D., Rochester.

"Supra-Pubic Prostatectomy; Demonstrating Operative Technic by Animated Drawings."—William E. Lower, M. D., Cleveland, O.; Associate Professor, Genito-Urinary Surgery, Western Reserve University.

Case Presentation: J. Henry Dowd, M. D., Buffalo. Discussion: George W. Cottis, M. D., Jamestown, and Thomas H. McKee, M. D., Buffalo.

"Causes of Goitre."—Martin B. Tinker, M. D., Ithaca, President of the Medical Society of the State of New York; Assistant Professor, Surgery, Cornell University. (Stereopticon).

Case Presentation: Frederick T. Munson, M. D., Friendship. Discussion: John M. Swan, M. D., Rochester; George F. Cott, M. D., Buffalo, and William D. Johnson, M. D., Batavia.

"Cancer of the Rectum."—William Francis Campbell, M. D., Brooklyn; Professor, Anatomy, Long Island College Hospital. (Stereopticon). Discussion: Descum C. McKenney, M. D., Buffalo; William R. Thompson, M. D., Warsaw, and Harvey R. Gaylord, M. D., Buffalo.

"Pituitary Body and Pituitarism."—James A. Gibson, M. D., Buffalo; Professor, Anatomy, University of Buffalo. (Stereopticon).

Case Presentation: F. Park Lewis, M. D., Buffalo. Discussion: G. Kirby Collier, M. D., Sonyea.

**The Elmira Clinical Society** met Sept. 20 at the residence of Dr. Clyde Corey who presented a paper on Infections of the Eye due to Poultices.

---

**The Chemung Co. Medical Society** held its regular meeting in Elmira Sept. 19 under the presidency of Dr. R. H. V. Dann. Dr. Geo. Case presented a paper on "When Tonsils should be removed;" Dr. Anna Stuart on Surgical Haemorrhage.

---

**The Elmira Academy of Medicine** held its regular meeting Sept. 6, under the presidency of Dr. LaRue Colegrove. Dr. R. P. Bush of Horseheads read a paper on The State and Public Health; Dr. R. B. Howland of Elmira on Infantile Paralysis.

---

**The Seventh District Branch of the Medical Society of the State of N. Y.** held its tenth annual meeting in Rochester, Sept. 28. The officers who hold over for the next year are as follows: President, William Mortimer Brown, M. D., Rochester; First Vice-President, Fred R. Driesbach, M. D., Dansville; Second Vice-President, Charles E. Doubleday, M. D., Penn Yan; Secretary, John F. Myers, M. D., Sodus; Treasurer, Alfred W. Armstrong, M. D., Canandaigua; Reception Committee, Howard L. Prince, M. D., Chairman; Owen E. Jones, M. D.; Willis E. Bowen, M. D.; John R. Booth, M. D.; William I. Dean, M. D.

The scientific session was as follows, luncheon being served by the Medical Society of the Co. of Monroe at the Genesee Valley Club:

Opening Address, William Mortimer Brown, M. D., Rochester.

Address by President of the Medical Society of the State of New York, Martin B. Tinker, M. D., Ithaca.

"Application of the So-Called New Wartime Antiseptics to the Surgery of Civil Life," Charles W. Hennington, M. D., Rochester.

"A Composite Osteoplastic Operation for Wide Congenital Clefts of the Palate," John B. Roberts, M. D., Philadelphia.

Discussion, John Marvin Ingersoll, M. D., Cleveland, Ohio.

"Experiences with the Bone Graft in the War Zone of France," illustrated with moving pictures and slides, Fred H. Albee, M. D., New York.

Discussion, Howard L. Prince, M. D., Rochester.

"Seventeen Years of Organized Malpractise Defence," James Taylor Lewis, New York.

"Epidemiology of Poliomyelitis," Harold L. Amoss, M. D., Rockefeller Institute, New York.

Discussion, George W. Goler, M. D., Rochester.

"The Prognosis in Infantile Paralysis," Robert W. Lovett, M. D., Boston.

Discussion, Brainerd Hunt Whitbeck, M. D., New York.

"Practical Methods for Conserving the Bad Risk Patient," George W. Crile, M. D., Cleveland, Ohio.

Discussion, Martin B. Tinker, M. D., Ithaca.

"Is Medicine a Business?" John Mumford Swan, M. D., Rochester.

Discussion, Henry Lyle Winter, M. D., Cornwall; Albert T. Lytle, M. D., Buffalo, and William B. Jones, M. D., Rochester.

---

**The Medical Association of Central N. Y.** will hold its 48th annual meeting in Buffalo, Thursday, Oct. 19.

